# The Impact of Synchronous Telehealth Services With a Digital Platform on Day-by-Day Home Blood Pressure Variability in Patients with Cardiovascular Diseases: Retrospective Cohort Study

**DOI:** 10.2196/22957

**Published:** 2022-01-10

**Authors:** Ying-Hsien Chen, Chi-Sheng Hung, Ching-Chang Huang, Jen-Kuang Lee, Jiun-Yu Yu, Yi-Lwun Ho

**Affiliations:** 1 Department of Internal Medicine National Taiwan University Hospital Taipei Taiwan; 2 Department of Business Administration College of Management National Taiwan University Taipei Taiwan

**Keywords:** blood pressure, variability, telehealth, hypertension, cardiovascular disease, chronic disease, heart, digital platform, cohort, management, intervention

## Abstract

**Background:**

Hypertension is associated with a large global disease burden with variable control rates across different regions and races. Telehealth has recently emerged as a health care strategy for managing chronic diseases, but there are few reports regarding the effects of synchronous telehealth services on home blood pressure (BP) control and variability.

**Objective:**

The objective of this study is to investigate the effect of synchronous telehealth services with a digital platform on home BP.

**Methods:**

This retrospective study was conducted by the Taiwan ELEctroHEALTH study group at the Telehealth Center of the National Taiwan University Hospital. We analyzed home BP data taken from 2888 patients with cardiovascular disease (CVD) enrolled in our telehealth program between 2009 to 2017. Of the 2888 patients with CVD, 348 (12.05%) patients who received home BP surveillance for ≥56 days were selected for BP analysis. Patients were stratified into three groups: (1) poorly controlled hypertension, (2) well-controlled hypertension, and (3) nonhypertension. The mean, SD, coefficient of variation (CV), and average real variability were calculated.

**Results:**

Telehealth interventions significantly and steadily reduced systolic blood pressure (SBP) in the poorly controlled hypertension group from 144.8.2±9.2 to 133.7±10.2 mmHg after 2 months (*P*<.001). BP variability reduced in all patients: SBP-SD decreased from 7.8±3.4 to 7.3±3.4 after 2 months (*P*=.004), and SBP-CV decreased from 6.3±2.5 to 5.9±2.6 after 2 months (*P*=.004). Event-free survival (admission) analysis stratified by SBP-SD showed longer time to first hospitalization for Q1 patients compared with Q4 patients (*P*=.02, odds ratio 2.15, 95% CI 1.18-3.89).

**Conclusions:**

Synchronous telehealth intervention may improve home BP control and decrease day-by-day home BP variability in patients with CVD.

## Introduction

According to data sourced from the noncommunicable disease (NCD) Risk Factor Collaboration, the number of adults worldwide with increased blood pressure (BP) was 1.13 billion in 2015, up from 594 million in 1975 [1]. As the number of adults with elevated BP levels continues to rise, hypertension is becoming a growing public health threat and an important global NCD. Globally, hypertension is considered a leading risk factor for both cardiovascular disease (CVD) and chronic kidney disease [2]. The relationship between the risks of CVD and BP can be continuous or log-linear [3]. Starting at BP levels of 115/75 mmHg, mortality risks associated with stroke, heart disease, or other CVDs are doubled with every 20 mmHg increase in systolic blood pressure (SBP) and every 10 mmHg increase in diastolic blood pressure (DBP) [3], while treatment to control BP can provide proportional reductions in risk that are independent of pretreatment BP levels [4].

The best hypertension management practices combine pharmacotherapy and nonpharmacological interventions, such as weight reduction, increased physical activity, the Dietary Approaches to Stop Hypertension (DASH) diet, potassium supplements, decreased sodium intake, and reduced alcohol consumption [5-7]. Despite widespread initiatives for treating hypertension and the availability of antihypertensive medications, more than 50% people in the United States have uncontrolled, elevated BP [8]. Among adults with hypertension in the United States, hypertension control rates are around 50%-60% for non-Hispanic Whites but less than 50% for non-Hispanic Black, Hispanic, and non-Hispanic Asian populations [9]. This phenomenon is primarily attributed to a lack of awareness and treatment, as well as suboptimal adherence to self-care [9,10]. It is recommended that structured and team-based interventions be paired with technology-based strategies rooted in health information to control and treat hypertension [6]. Telehealth strategies, such as telemedicine, eHealth, and mHealth, are innovative tools for BP control. Meta-analyses of randomized controlled trials (RCTs) using different telehealth interventions have demonstrated greater BP reductions and a larger proportion of patients achieving BP control [11,12]. However, the involvement of various telehealth interventions within a single strategy can lead to inconsistent clinical effects. Anker et al [13] proposed a classification system enabling comparison between various telehealth strategies for the four different generations of telemedicine based on data transfer procedures, the analytical ability of the telehealth system, and the degree of integration with patient primary health care structures. The telehealth service from the National Taiwan University Hospital (NTUH), Taipei, Taiwan, has provided a fully integrated remote management system with constant analytical and decision-making support for full therapeutic authority outside office hours since 2009. This service model fulfills the criteria for the fourth-generation telehealth service/program proposed by Anker et al [13]. As evidence from use of a fourth-generation telehealth program for home BP control and home BP variability is currently lacking, we decided to analyze patient data collected through the Telehealth Center of the NTUH to determine the impacts of our telehealth intervention services on home BP control and home BP variability. The objective of our study was to investigate the effect of synchronous telehealth services with a digital platform on home BP.

## Methods

### Study Design

This was a single-center retrospective cohort study approved by the institutional review board of the NTUH and conducted by the Taiwan ELEctroHEALTH (TELEHEALTH) study group. We obtained written informed consent from all participants for telehealth services.

### Telehealth Services

Since 2009, the Telehealth Center of the NTUH has provided telehealth services for patients with CVD, including both patients with multiple CVD risk factors and patients with established CVDs, such as coronary artery disease, myocardial infarction, congestive heart failure, arrhythmia, and other surgical or congenital heart conditions. Detailed descriptions of enrollment criteria and the scope of telehealth services have been reported previously [14].

Our telehealth services encompassed surveillance of biometric data, discussions between patients and the Telehealth Center, and full therapeutic authority and suggestions from case managers and cardiologists 24 hours a day. Patients with CVD were required to measure vital biometric data (single-lead electrocardiography, BP, pulse rate [PR], finger-stick glucose, and oxygen saturation) at home daily or on demand. Data were then transmitted to a cloud database for review and evaluation by nurse case managers or physicians who were able to access the data via an internet-based interactive platform and provide instant advice according to the clinical conditions of each patient [14-17]. The internet-based interactive platform used by the Telehealth Center was developed by the Graduate Institute of Biomedical Electronics and Bioinformatics, National Taiwan University, Taiwan [18]. The ultimate goal of the telehealth program was to bridge the gap between acute care and home care and also to emphasize education, enhance self-awareness, improve self-care skills, strengthen prevention, and facilitate early detection of clinical deterioration.

### Patient Selection

To evaluate day-by-day BP changes over a period of 8 weeks, patients with CVD who had received telehealth services and recorded daily home BP measurements consecutively for more than 56 days (8 weeks) during 2009 to 2017 were retrospectively selected for analysis. Patients who were enrolled in the telehealth program for less than 56 days or those who failed to transmit daily home BP measurements were excluded from our analysis. No statistical power calculation was conducted prior to the study. The sample size was based on the available data. All statistical tests were determined after examination of available data.

### Home BP

We compiled SBP, DBP, and PR data from all patients to serve as explanatory variables. Mean blood pressure (MBP) values were calculated using the formula MBP = [SBP + (2 × DBP)]/3. We calculated the daily home MBP values for every selected patient from days 1 to 56 and then calculated baseline home BP by averaging the first 3 days (days 1-3) of home BP values. Thereafter, average posttelehealth BP values were calculated in 14-day (2-week) intervals: values for week 2 were calculated by averaging BP values from days 4 to 14, values for week 4 were calculated by averaging BP values from days 15 to 28, values for week 6 were calculated by averaging BP values from days 29 to 42, and values for week 8 were calculated by averaging BP values from days 43 to 56. Calculations were conducted in the same manner for SBP, DBP, MBP, and PR data.

### Home BP Variability

Home BP variability values were calculated by SD, coefficient of variation (CV), and average real variability (ARV) [19]. Home BP variability was calculated in 14-day (2-week) intervals: baseline values were derived from days 1 to 14, weeks 3-4 values from days 15 to 28, weeks 5-6 values from days 29 to 42, and weeks 7-8 values from days 43 to 56. Calculations were conducted in the same manner for SBP and DBP data.

### Group Analysis

We divided patients into the following groups based on clinical diagnosis and BP data at baseline: (1) poorly controlled hypertension group, which included hypertension patients with baseline home SBP ≥135 mmHg or home DBP ≥85 mmHg according to European Society of Cardiology (ESC) guideline definitions [7]; (2) well-controlled hypertension group, which included hypertension patients with baseline home SBP <135 mmHg and home DBP <85 mmHg; and (3) nonhypertension group, which included patients who had not previously been diagnosed with hypertension. All group analyses were determined based on examinations of available data.

### Outcome Measurement and Follow-Up

Mean values and variability indicators for home BP levels after telehealth services were compared with baseline values (at prespecified durations after telehealth services) at 2-week intervals. Clinical outcomes regarding the time to first hospitalization were censored in selected patients with CVD with a mean follow-up period of 22 months.

### Statistical Analysis

Continuous data are presented as the mean±SD. Discrete data are given as counts and percentages. Paired *t*-tests were used to analyze changes in different data sets over time for the same individuals compared with baseline. Between-group comparisons of discrete indicators at baseline (gender proportions, comorbidities, and medications) were analyzed using a chi-square test, while between-group comparisons of continuous indicators at baseline (age, average BP measurements per day, hemoglobin A_1c_ (HbA_1c_) levels, creatinine levels, and total cholesterol) were calculated using an ANOVA test.

We used paired *t*-tests to compare differences in BP values between patient groups. Basic assumptions for paired *t*-tests were verified prior to conducting these analyses. Independently observed continuous BP values were set as our dependent variable. SBP values <65 mmHg or >250 mmHg and DBP values <40 mmHg or >150 mmHg were excluded to avoid outliers. Two-week average BP values were found to be normally distributed based on graphical presentations of QQ plots and histograms. One-way ANOVA and chi-square tests were used to compare differences in baseline characteristics between patient groups. Basic assumptions for one-way ANOVA and chi-square tests were verified prior to conducting these analyses. One-way ANOVA was used for continuous data, such as age, average BP measurements per day, HbA_1c_, creatinine, and total cholesterol. All data adhered to ANOVA assumptions of normality (based on QQ plots), homoscedasticity (based on scatterplot of residuals), no multicollinearity (based on correlation data and variance inflation factor), and independence of measurements. Chi-square tests were used for independent categorical data such as gender, comorbidities, and medication.

A two-sided *P* value of <0.05 was considered statistically significant. Kaplan-Meier analysis was used to compare time to first hospitalization for different groups, and the log-rank test (pairwise over strata) was used to detect differences between survival curves. Patients were censored if they were not hospitalized more than 36 months after entry into our telehealth program or if there were insufficient data to indicate that they were hospitalized during this period. IBM SPSS for Windows, version 22.0, (SPSS Inc., Chicago, IL, USA) was used for statistical analyses.

## Results

### Descriptive Statistics

From 2009 to 2017, a total of 2888 patients with CVD received synchronous telehealth services and delivered a total of 678,596 BP data sets over varying lengths of time. Of these, 2540 patients had insufficient data over a 56-day period and were excluded from the study. The majority of missing data in the cohort was due to participants forgetting to take BP measurements or leaving the program before 56 days. A total of 348 patients (mean age 67.6±14.6 years; 232 [66.7%] males) who recorded daily home BP measurements for more than 56 days were selected for BP analysis. During their 56-day period of telehealth care (days 1-56), 383,400 BP data sets were transmitted for telehealth surveillance.

The patients were further stratified into the (1) poorly controlled hypertension group (n=81), (2) well-controlled hypertension group (n=125), and (3) nonhypertension group (n=142) according to prespecified definitions. Baseline demographics and medications before telehealth care are summarized in Table 1. In the poorly controlled hypertension group, the mean age was significantly higher (*P*=.001) and patients had more comorbidities and higher rates of renin-angiotensin system (RAS) blocker usage compared with the other groups.

**Table 1:** Baseline characteristics of telehealth service patients.

Characteristics, diseases, and medications		Poorly controlled hypertension	Well-controlled hypertension	Nonhypertension	Total patients	*P* value
**Characteristics**						
	Patients	n=81	n=125	n=142	N=348	—^a^
	Age (years), mean±SD	72.0±12.2	68.1±13.8	64.6±15.8	67.6±14.6	.001
	Male, n (%)	52 (64%)	79 (63.2%)	101 (71.1%)	232 (66.7%)	.34
**Diseases**						
	Hypertension, n (%)	81 (100%)	125 (100%)	0 (0.0%)	183 (52.6%)	<.001
	Heart failure, n (%)	15 (19%)	29 (23.2%)	39 (27.5%)	83 (23.9%)	.31
	Diabetes mellitus, n (%)	37 (46%)	55 (44.0%)	18 (12.7%)	110 (31.6%)	<.001
	Hyperlipidemia, n (%)	24 (30%)	64 (51.2%)	30 (21.1%)	118 (33.9%)	<.001
	Myocardial infarction, n (%)	9 (11%)	17 (13.6%)	18 (12.7%)	44 (12.6%)	.87
	Coronary artery disease, n (%)	37 (46%)	72 (57.6%)	57 (40.1%)	166 (47.7%)	.02
	Stroke, n (%)	6 (7%)	23 (18.4%)	6 (4.2%)	35 (10.1%)	<.001
	End-stage renal failure, n (%)	16 (20%)	14 (11.2%)	4 (2.8%)	34 (9.8%)	<.001
	Atrial fibrillation, n (%)	10 (12%)	24 (19.2%)	26 (18.3%)	60 (17.2%)	.40
	Average BP^b^ measurements per day	2.3±0.9	2.2±1.1	2.1±0.7	2.2±0.9	.21
	HbA_1c_^c^ (%), mean±SD	6.5±1.0	6.4±1.0	6.2±1.1	6.3±1.0	.05
	Creatinine^d^ (mg/dL), mean±SD	1.8±2.3	1.3±1.4	1.3±1.2	1.4±1.5	.06
	Total cholesterol (mg/dL), mean±SD	166.6±43.9	158.9±34.8	159.6±36.9	160.9±37.9	.33
**Medications**						
	Calcium channel blocker, n (%)	4 (5%)	12 (9.6%)	8 (5.6%)	24 (6.9%)	.32
	Diuretics, n (%)	18 (22%)	33 (26.4%)	24 (16.9%)	75 (21.6%)	.17
	Beta blocker, n (%)	16 (20%)	29 (23.2%)	32 (22.5%)	77 (22.1%)	.84
	RAS^e^ blocker, n (%)	25 (31%)	48 (38.4%)	23 (16.2%)	96 (27.6%)	.001

^a^—: not applicable.

^b^BP: blood pressure.

^c^HbA_1c_: hemoglobin A_1c_.

^d^Patients with renal failure under dialysis were excluded.

^e^RAS: renin-angiotensin system. The RAS blocker includes angiotensin-converting enzyme inhibitors, angiotensin receptor blockers, and angiotensin receptor neprilysin inhibitor.

### Home BP Change After Telehealth Service

In the poorly controlled hypertension group, BP significantly and constantly decreased after 2 weeks of telehealth service. During the 8 weeks of telehealth service, the mean SBP decreased from baseline 144.8±9.2 mmHg to 133.7±10.2 mmHg (*P*<.001) and the mean DBP also decreased from baseline 77.7±12.7 mmHg to 72.9±12.1 mmHg (*P*<.001).

In the well-controlled hypertension group, a gradual increment in BP was observed. During the 8 weeks of telehealth service, the mean SBP increased from baseline 119.7±9.8 mmHg to 124.1±11.3 mmHg (*P*<.001) and the mean DBP increased from baseline 71.3±9.9 mmHg to 73.0±9.4 mmHg (*P*=.009). A slight increment in home BP (1.7 mmHg in SBP and 1.6 mmHg in DBP) was also observed in the nonhypertension group during the 8 weeks of telehealth service (Table 2). The effect of telehealth services on the PR and MBP in different subgroups is provided in Multimedia Appendix 1. The impact of telehealth services on BP values in all patients is provided in Multimedia Appendix 2.

**Table 2:** Change in BP^a^ during the period of telehealth service by subgroup.

BP measures		Baseline (days 1-3)	Week 2 (days 4-14)	Week 4 (days 15-28)	Week 6 (days 29-42)	Week 8 (days 43-56)
**Poorly controlled hypertension group**						
	SBP^b^ (mmHg), mean±SD	144.8±9.2	140.7±10.0	137.1±12.0	136.0±11.8	133.7±10.2
	*P* value^c^	—^d^	<.001	<.001	<.001	<.001
	DBP^e^, mmHg	77.7±12.7	76.0±11.0	74.8±12.2	73.9±11.9	72.9±12.1
	*P* value	—	.01	.002	<.001	<.001
**Well-controlled hypertension group**						
	SBP (mmHg), mean±SD	119.7±9.8	121.9±10.6	123.1±10.7	123.3±11.0	124.1±11.3
	*P* value	—	.004	<.001	<.001	<.001
	DBP (mmHg), mean±SD	71.3±9.9	72.0±9.4	72.8±9.5	72.8±9.2	73.0±9.4
	*P* value	—	.11	.006	.02	.009
**Nonhypertension group**						
	SBP (mmHg), mean±SD	115.6±11.0	115.5±11.7	116.0±11.6	116.8±11.5	117.3±11.6
	*P* value	—	.81	.56	.13	.04
	DBP (mmHg), mean±SD	68.6±8.8	69.1±8.4	69.1±9.1	70.0±9.0	70.2±9.4
	*P* value	—	.25	.35	.02	.01

^a^BP: blood pressure.

^b^SBP: systolic blood pressure.

^c^Data were expressed as the mean±SD and were compared with baseline.

^d^—: not applicable.

^e^DBP: diastolic blood pressure.

### Change in Home BP Variability After Telehealth Service

Home day-by-day BP variability in different subgroups decreased following telehealth services (Table 3). In the poorly controlled hypertension group, SBP-SD decreased from 9.2±4.1 mmHg (baseline) to 8.2±3.5 mmHg during weeks 5-6 (*P*=.04) and stayed constant thereafter (variability for weeks 7-8 did not rise significantly compared with variability for weeks 5-6). DBP-SD decreased from 5.7±2.5 mmHg (baseline) to 4.9±1.8 mmHg during weeks 5-6 (*P*=.02) and remained stable thereafter. We found no significant changes with regard to SBP or DBP variability measured by the CV or ARV.

In the well-controlled hypertension group, SBP-CV during weeks 7-8 was 6.0%±2.6%, which was significantly lower than baseline SBP-CV (6.5%±2.3%, *P*=.02), and DBP-CV during weeks 5-6 was 6.9%±3.0%, which was also significantly lower than baseline DBP-CV (7.5%±2.9%, *P*=.01).

In the nonhypertension group, a significant decrease in DBP-SD was observed during weeks 3-4 compared with baseline.

**Table 3:** Change in BP^a^ variability during the period of telehealth service by subgroup.

BP measures		Baseline (days 1-14)	Weeks 3-4 (days 15-28)	Weeks 5-6 (days 29-42)	Weeks 7-8 (days 43-56)
**Poorly controlled hypertension group**					
	SBP^b^-SD (mmHg), mean±SD	9.2±4.1	8.5±3.5	8.2±3.5	8.3±4.1
	*P* value^c^	—^d^	.14	.04	.09
	SBP-CV^e^ (%), mean±SD	6.5±2.8	6.2±2.6	6.0±2.5	6.2±3.0
	*P* value	—	.39	.16	.40
	SBP-ARV^f^ (mmHg), mean±SD	8.6±4.0	8.7±3.9	8.6±4.1	8.7±4.0
	*P* value	—	.84	.86	.88
	DBP^g^-SD (mmHg), mean±SD	5.7±2.5	5.3±3.3	4.9±1.8	5.0±2.1
	*P* value	—	.43	.02	.06
	DBP-CV (%), mean±SD	7.4±3.1	7.3±4.8	6.8±2.7	7.0±2.9
	*P* value	—	.81	.11	.28
	DBP-ARV (mmHg), mean±SD	5.3±2.5	5.5±3.1	5.1±1.9	5.3±2.3
	*P* value	—	.55	.49	.98
**Well-controlled hypertension group**					
	SBP-SD (mmHg), mean±SD	8.0±3.2	7.7±3.7	7.5±3.0	7.5±3.3
	*P* value	—	.46	.15	.08
	SBP-CV (%), mean±SD	6.5±2.3	6.2±2.8	6.1±2.3	6.0±2.6
	*P* value	—	.28	.08	.02
	SBP-ARV (mmHg), mean±SD	8.0±3.5	7.9±3.2	8.0±3.5	8.1±3.6
	*P* value	—	.75	.90	.70
	DBP-SD (mmHg), mean±SD	5.3±1.9	5.3±2.4	5.0±2.1	5.3±3.1
	*P* value	—	.99	.05	.99
	DBP-CV (%), mean±SD	7.5±2.9	7.3±3.2	6.9±3.0	7.4±4.8
	*P* value	—	.50	.01	.83
	DBP-ARV (mmHg), mean±SD	5.3±2.2	5.4±2.5	5.2±2.3	5.8±3.2
	*P* value	—	.67	.69	.08
**Nonhypertension group**					
	SBP-SD (mmHg), mean±SD	6.9±2.9	6.6±2.9	6.7±3.1	6.6±2.8
	*P* value	—	.17	.29	.15
	SBP-CV (%), mean±SD	6.0±2.4	5.7±2.3	5.7±2.6	5.6±2.3
	*P* value	—	.11	.19	.08
	SBP-ARV (mmHg), mean±SD	6.7±2.9	7.0±3.2	7.2±3.1	7.0±3.3
	*P* value	—	.23	.09	.14
	DBP-SD (mmHg), mean±SD	4.9±2.2	4.6±2.0	4.7±2.4	4.9±2.3
	*P* value	—	.05	.27	.96
	DBP-CV (%), mean±SD	7.2±3.2	6.7±3.0	6.7±3.3	7.2±3.6
	*P* value	—	.05	.13	.88
	DBP-ARV (mmHg), mean±SD	4.8±2.2	4.8±2.1	5.0±2.3	5.1±2.4
	*P* value	—	.96	.56	.19

^a^BP: blood pressure.

^b^SBP: systolic blood pressure.

^c^Data were expressed as the mean±SD and were compared with baseline.

^d^—: not applicable.

^e^CV: coefficient of variation.

^f^ARV: average real variability.

^g^DBP: diastolic blood pressure.

The effect of telehealth services on day-by-day home BP variability in all patients is provided in Table 4. Telehealth services significantly improved SBP variability (SD and CV) during weeks 3-4 and remained constant during weeks 5-6 and weeks 7-8. DBP variability (SD and CV) was also significantly improved at weeks 5-6 compared with baseline.

**Table 4:** Change in BP^a^ variability during the period of telehealth service in all patients (N=348).

BP measures	Baseline (days 1-14)	Weeks 3-4 (days 15-28)	Weeks 5-6 (days 29-42)	Weeks 7-8 (days 43-56)
SBP^b^-SD, mmHg	7.8±3.4	7.5±3.4	7.3±3.2	7.3±3.4
*P* value^c^	—^d^	.04	.008	.004
SBP-CV^e^, %	6.3±2.5	6.0±2.6	5.9±2.5	5.9±2.6
*P* value	—	.04	.009	.004
SBP-ARV^f^, mmHg	7.6±3.5	7.7±3.4	7.8±3.5	7.8±3.6
*P* value	—	.54	.38	.35
DBP^g^-SD, mmHg	5.2±2.2	5.0±2.5	4.8±2.2	5.1±2.6
*P* value	—	.11	.003	.31
DBP-CV, %	7.4±3.1	7.1±3.6	6.8±3.1	7.2±4.0
*P* value	—	.12	.002	.46
DBP-ARV, mmHg	5.1±2.3	5.2±2.5	5.1±2.2	5.4±2.7
*P* value	—	.55	.85	.06

^a^BP: blood pressure.

^b^SBP: systolic blood pressure.

^c^Data were expressed as the mean±SD and were compared with baseline.

^d^—: not applicable.

^e^CV: coefficient of variation.

^f^ARV: average real variability.

^g^DBP: diastolic blood pressure.

### Time to First Hospitalization After Telehealth Services

**Figure 1:**
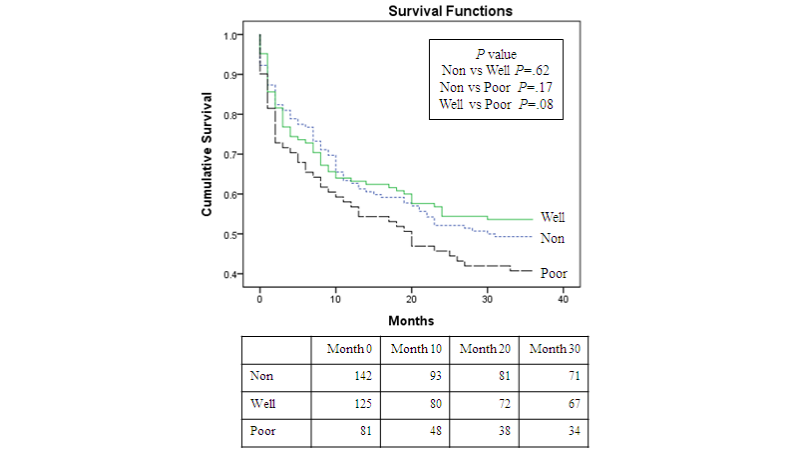
Kaplan-Meier survival estimates for time to first hospitalization stratified by hypertension type (poor: poorly controlled hypertension; well: well-controlled hypertension; non: nonhypertension).

No significant differences were found between the three subgroups in terms of time to first hospitalization (*P*=.20; Figure 1) during a mean follow-up period of 22 months.

**Figure 2:**
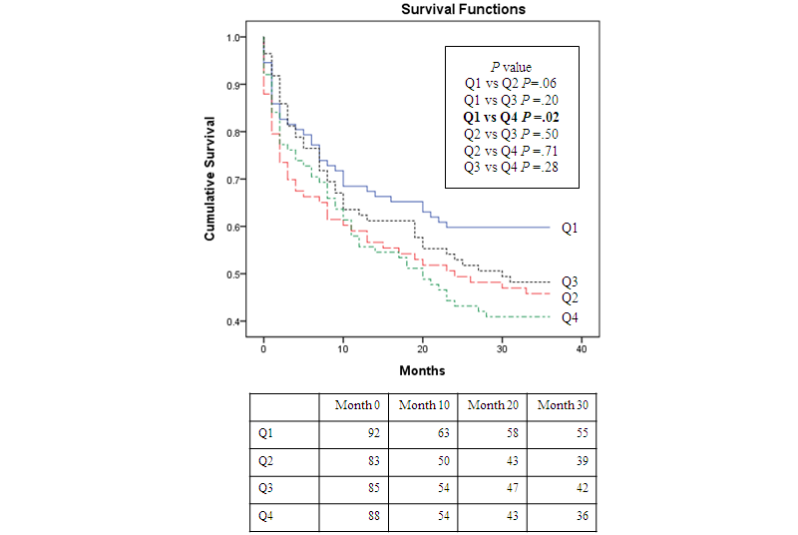
Kaplan-Meier survival estimates for time to first hospitalization across quartiles of day-by-day variability, SD of systolic blood pressure at baseline. Q1 to Q4 indicate ascending quartiles: (Q1: <5.5, Q2: 5.5-7.1, Q3: 7.1-9.7, Q4: >9.7).

We further stratified these patients according to baseline SBP-SD quartiles (Q1: <5.5; Q2: 5.5-7.1; Q3: 7.1-9.7; and Q4: >9.7), and a significant difference in time to first hospitalization was observed between Q1 and Q4 SBP-SD variability groups (*P*=.02, odds ratio 2.15, 95% CI 1.18-3.89; Figure 2).

## Discussion

### Principal Findings

The findings from this retrospective study suggest that synchronous telehealth intervention may improve home BP control and decrease home BP variability in patients with CVD.

For patients with poorly controlled hypertension, our telehealth intervention demonstrated a significant BP reduction after only 2 weeks of service. BP reductions were continuous and remained steady throughout the 8-week period of telehealth services, achieving an absolute 11.1 mmHg SBP reduction and a 4.8 mmHg DBP reduction after 8 weeks of telehealth intervention. For patients without hypertension or with well-controlled hypertension, the telehealth intervention helped to normalize home BP, possibly by avoiding overtreatment and hypotension. The telehealth intervention also decreased BP variability after 4 weeks of telehealth services, and this effect remained constant hereafter and may be observed across the three different subgroups.

### Telehealth Services Improved Home BP Control

A higher SBP and DBP are known to be associated with increased risk of CVD [20], and high BP variability is associated with cardiovascular end-organ damage, renal outcomes, and increased risk of incident CVD [19,21,22]. Our study therefore demonstrates the value of telehealth services in controlling both home BP and home BP variability, which can potentially help to prevent future occurrence of CVD events.

Our previous study found that all-cause admission rates and all-cause hospital stay durations of patients with CVD decrease following synchronous telehealth interventions [14]. When compared with usual care, synchronous telehealth interventions were found to result in better cost-effectiveness [15] and were also proven to be associated with lower all-cause mortality [16]. It is speculated that these proven clinical benefits stemmed from continuous improvement in the control of chronic conditions, including BP control, which in turn led to reduced mortality. The results from this retrospective study provide additional evidence to support this speculation.

### Telehealth Services Normalized Home BP

During the 8 weeks of continuous telehealth intervention, home BP values significantly increased in the well-controlled hypertension group (mean SBP increased from 119.7±9.8 to 124.1±11.3 mmHg) and the nonhypertension group (SBP increased from 115.6±11.0 to 117.3±11.6 mmHg). This paradoxical increase in home BP should be considered a normalization of BP via telehealth interventions, which avoid overtreatment and hypotension. Patients in the nonhypertension group still received calcium channel blockers, beta blockers, diuretics, or RAS blockers for clinical indications other than hypertension, such as arrhythmia or heart failure; this patient population was considered highly susceptible to hypotension during treatment, and it may be that our telehealth interventions helped to prevent hypotensive events. This is worth noting, as a lower BP is associated with a worse prognosis for patients with heart failure [23].

The Systolic Blood Pressure Intervention Trial (SPRINT) study [24] aimed for an SBP goal of <120 mmHg. In the intensive treatment group, a marked decrease in the SBP was accompanied by a marked increase in the incidence of side effects, such as hypotension, syncope, electrolyte abnormalities, and kidney injures; similar results were also observed in the Action to Control Cardiovascular Risk in Diabetes (ACCORD) study [25].

Among treated hypertensive patients, SBP values of <110 mmHg are associated with potential serious falls and syncope [26]. Additionally, hypotension or masked hypotension occurs frequently in older hypertensive adults undergoing treatment [27,28], and low daytime SBP values are also known to be associated with a greater level of cognitive decline in these older patients [29]. Hypotension in hypertensive elderly patients undergoing treatment is an important red flag that makes it unclear whether these patients actually benefit from BP control. Out-of-office BP (including ambulatory BP monitoring or home BP monitoring) is specifically recommended as a method for identifying hypotension side effects during treatment [7]. Synchronous telehealth interventions not only help to identify hypotension side effects but may also exert an additional role in avoiding overtreatment and preventing hypotension.

### Other Telehealth Interventions for BP

The American Heart Association (AHA) recommends the use of out-of-office BP measurements paired with telehealth counseling and titration of BP-lowering medication to assess treatment response, improve adherence, and enhance BP control [6].

Meta-analyses of RCTs have shown that highly heterogeneous telehealth intervention models ranging from computer-based support systems to programs led by medical staff can improve BP control and achieve BP normalization in a larger proportion of patients [11,12]. Omboni et al [11] analyzed 23 RCTs (7037 patients) and found that telemonitoring of home BP demonstrates an improvement over office SBP by 4.7 mmHg and office DBP by 2.5 mmHg compared with usual care [11]. The Telemonitoring and/or Self-Monitoring of Blood Pressure in Hypertension (TASMINH4) trial, an unmasked RCT study that compared self-monitored BP with and without telemonitoring in patients with poorly controlled BP, failed to demonstrate any BP control benefits associated with telemonitoring. However, the study was conducted in 142 general practices (GPs) throughout the United Kingdom, which provided a simple and free SMS text-based telemonitoring service. Participants in the telemonitoring group received an SMS message on a weekly basis containing a report of MBP values. Overly high or low readings triggered an alert, which was sent to participants but not GPs, and attending clinicians reviewed the readings on a monthly basis [30]. This asynchronous telemonitoring design meant that medical advice was often delayed for several weeks and was perhaps why the results of the study failed to find benefits associated with telemonitoring. By contrast, the Telehealth Center of the NTUH offers a fourth-generation telehealth program that is fully integrated with remote management systems and synchronous data transfers that allow for constant analytical support backed by full therapeutic authority and decision making from physicians outside office hours [13]. The TASMINH4 trial provides a sharp contrast to our work and confirms our belief that synchronous telehealth care that preserves people-to-people communication is the key for successful telehealth interventions. Our study results support the use of synchronous telehealth services to control home BP in patients with CVD.

### Baseline BP Variability Predicts Clinical Outcomes

In our study, cumulative incidence for time to first hospitalization differed across baseline SBP-SD quartiles, implying that baseline BP variability may have some prognostic value. The Ohasama study [21] calculated day-by-day BP variability in 2455 residents in Ohasama, Japan, who measured home BP once every morning for 26 days. Over a median follow-up period of 11.9 years, an increase in SBP variability of +1 between-subject SD was found to be associated with increased hazard ratios for cardiovascular death (1.27; *P*=.002) and stroke mortality (1.41; *P*=.001). The Japan Morning Surge - Home Blood Pressure (J-HOP) study [19] calculated day-by-day BP variability in 4231 participants who measured home BP each morning and evening over a 14-day period. After 4 years of follow-up, greater day-by-day home SBP variability was associated with increased risk of incident CVD and cardiovascular end-organ damage. Both the Ohasama study and the J-HOP study concluded that baseline or short periods of day-by-day home BP variability provide useful clinical information for assessing future cardiovascular risk. In terms of physiological conditions, BP variations occur on a beat-by-beat basis and may represent a complex homeostatic control or response to neural, humoral, vascular, environmental, behavioral, and emotional stimuli [31]. Day-by-day home BP variability is not constant and can be modified: our study demonstrated that synchronous telehealth interventions can significantly decrease day-by-day BP variability. To the best of our knowledge, this is the first study that demonstrates the value of synchronous telehealth services in improving day-by-day BP variability. Future studies are warranted to determine whether declines in day-by-day BP variability could prevent future CVD occurrence.

### Different Scales of BP Variability

Previous longitudinal and observational studies have shown that different types of BP variability determined by the duration of analysis can represent differing prognostic relevance for CVD and renal outcomes [22]. Durations range from very short term (beat-to-beat), short term (over 24 hours), mid-term (day-to-day), long term (visit-to-visit < 5 years), and very long term (visit-to-visit ≥ 5 years). Analysis over all durations provide a consistent message showing that higher BP variability predicts worse clinical outcomes. Higher mid-term BP variability was associated with higher rates of subclinical organ damage, stroke, myocardial infarction, cardiovascular mortality, all-cause mortality, microabuminuria, and lower estimated glomerular filtration rate [22]. The mid-term BP variability analyzed in our study showed that the cumulative incidence for time to first hospitalization is higher in patients with higher baseline BP variability, although the cumulative incidence for time to first hospitalization did not differ across subgroups stratified by hypertension status. This result may be attributed to the effects of synchronous telehealth interventions that improved BP control over time, particularly for the poorly controlled hypertension group.

The causative mechanism between BP variability and clinical outcomes is still unclear. Kario et al [32] proposed a systemic hemodynamic atherothrombotic syndrome theory that hypothesized that BP variability is caused by synergistic resonance, and demonstrated that BP exhibits different variabilities with different time phases (beat-by-beat, diurnal, day-by-day, visit-to-visit, seasonal, and yearly). Synergistic resonance of BP variabilities over different time phases can generate hemodynamic surges and trigger a vicious cycle of hemodynamic stress, which can lead to organ damage and ultimately advance to CVD.

### Limitations

There were several limitations in our study. First, the study was a non-RCT, retrospective study with no comparable usual-care group. Second, although our follow-up period continued for 22 months, most patients with CVD did not receive parallel continuous telehealth services. Third, the study included heterogenous patients with CVD both with and without hypertension. To clarify specific issues, these patients were further stratified into subgroups, thus lowering the patient numbers in each subgroup. Fourth, adjustment of medication data was lacking in our study.

### Conclusion

Synchronous telehealth interventions may improve home BP control and decrease day-by-day home BP variability in patients with CVD.

## Appendix

Multimedia Appendix 1 Table S1. Change in mean blood pressure and pulse rate during the period of telehealth in subgroups.

Multimedia Appendix 2 Table S2. Change in blood pressure during the period of telehealth in all patients.

## Abbreviations

ACCORD: Action to Control Cardiovascular Risk in Diabetes
ARV: average real variability
BP: blood pressure
CV: coefficient variability
CVD: cardiovascular disease
DBP: diastolic blood pressure
ESC: European Society of Cardiology
HbA_1c_: hemoglobin A_1c_
J-HOP: Japan Morning Surge - Home Blood Pressure
MBP: mean blood pressure
NCD: noncommunicable disease
NTUH: National Taiwan University Hospital
PR: pulse rate
RCT: randomized controlled trial
SBP: systolic blood pressure
SD: standard deviation
SPRINT: Systolic Blood Pressure Intervention Trial
TELEHEALTH: Taiwan ELEctroHEALTH
