# Supplementary material for: The Impact of Synchronous Telehealth Services With a Digital Platform on Day-by-Day Home Blood Pressure Variability in Patients with Cardiovascular Diseases: Retrospective Cohort Study
Source: J Med Internet Res. 2022 Jan 10;24(1):e22957. doi: 10.2196/22957 (PMC8787660; doi:10.2196/22957)
Supplement: Multimedia Appendix 1 [file jmir_v24i1e22957_app1.docx]

Table S1. Change in mean blood pressure and pulse rate during the period of telehealth in subgroups.

| Telehealth period ^a^ | Baseline | Week 2 | Week 4 | Week 6 | Week 8 |
| --- | --- | --- | --- | --- | --- |
| Poorly-controlled hypertension |  |  |  |  |  |
| MBP, mmHg | 100.1±9.7 | 96.7±8.4 | 95.6±10.0 | 94.6±9.4 | 93.2±9.6 |
| *P* value |  | <.001 | <.001 | <.001 | <.001 |
| PR, BPM | 74.8±13.1 | 74.0±11.7 | 73.7±11.1 | 73.6±11.1 | 73.2±10.9 |
| *P* value |  | .20 | .18 | .22 | .13 |
| Well-controlled hypertension |  |  |  |  |  |
| MBP, mmHg | 87.4±8.0 | 88.6±8.2 | 89.6±8.5 | 89.6±8.2 | 90.0±8.1 |
| *P* value |  | .02 | <.001 | .001 | <.001 |
| PR, BPM | 74.8±12.7 | 74.2±11.6 | 73.4±11.9 | 74.1±11.9 | 73.4±11.4 |
| *P* value |  | .32 | .06 | .43 | .11 |
| Non-hypertension |  |  |  |  |  |
| MBP, mmHg | 84.3±8.1 | 84.5±8.2 | 84.7±8.6 | 85.6±8.5 | 85.9±8.9 |
| *P* value |  | .54 | .38 | .03 | .01 |
| PR, BPM | 73.9±12.2 | 73.6±11.2 | 72.5±11.4 | 72.1±11.0 | 72.0±10.5 |
| *P* value |  | .46 | .05 | .03 | .04 |

a Baseline: day 1 to day 3, Week 2: day 4 to day 14, Week 4: day 15 to day 28, Week 6: day 29to day 42, Week 8: day 43 to day 56.

b Data were expressed as mean± SD, and were compared with the baseline.

Abbreviation: SBP: systolic blood pressure, DBP: diastolic blood pressure, MBP: mean blood pressure, PR: pulse rate, BPM: beats per minute
