# Supplementary material for: The Impact of Synchronous Telehealth Services With a Digital Platform on Day-by-Day Home Blood Pressure Variability in Patients with Cardiovascular Diseases: Retrospective Cohort Study
Source: J Med Internet Res. 2022 Jan 10;24(1):e22957. doi: 10.2196/22957 (PMC8787660; doi:10.2196/22957)
Supplement: Multimedia Appendix 2 [file jmir_v24i1e22957_app2.docx]

Table S2. Change in blood pressure during the period of telehealth in all patients

| Telehealth period ^a^ | Baseline | Week 2 | Week 4 | Week 6 | Week 8 |
| --- | --- | --- | --- | --- | --- |
| Total patients |  |  |  |  |  |
| SBP, mmHg | 123.9±15.5 | 123.7±14.7 | 123.5±14.0 | 123.6±13.6 | 123.6±12.9 |
| *P* value |  | .62 | .50 | .67 | .66 |
| DBP, mmHg | 71.7±10.8 | 71.8±9.8 | 71.7±1.3 | 71.9±9.9 | 71.8±10.2 |
| *P* value |  | .77 | .82 | .57 | .71 |
| MBP, mmHg | 89.1±10.5 | 89.1±9.6 | 89.0±9.8 | 89.1±9.3 | 89.1±9.2 |
| *P* value |  | .96 | .85 | .88 | .98 |
| PR, BPM | 74.4±12.6 | 73.9±11.5 | 73.1±11.2 | 73.2±11.4 | 72.8±10.9 |
| *P* value |  | .09 | .003 | .01 | .003 |

a Baseline: day 1 to day 3, Week 2: day 4 to day 14, Week 4: day 15 to day 28, Week 6: day 29 to day 42, Week 8: day 43 to day 56.

b Data were expressed as mean± SD, and were compared with the baseline.

Abbreviation: SBP: systolic blood pressure, DBP: diastolic blood pressure, MBP: mean blood pressure, PR: pulse rate, BPM: beats per minute
